# Shared genetic background between children and adults with attention deficit/hyperactivity disorder

**DOI:** 10.1038/s41386-020-0664-5

**Published:** 2020-04-12

**Authors:** Paula Rovira, Ditte Demontis, Cristina Sánchez-Mora, Tetyana Zayats, Marieke Klein, Nina Roth Mota, Heike Weber, Iris Garcia-Martínez, Mireia Pagerols, Laura Vilar-Ribó, Lorena Arribas, Vanesa Richarte, Montserrat Corrales, Christian Fadeuilhe, Rosa Bosch, Gemma Español Martin, Peter Almos, Alysa E. Doyle, Eugenio Horacio Grevet, Oliver Grimm, Anne Halmøy, Martine Hoogman, Mara Hutz, Christian P. Jacob, Sarah Kittel-Schneider, Per M. Knappskog, Astri J. Lundervold, Olga Rivero, Diego Luiz Rovaris, Angelica Salatino-Oliveira, Bruna Santos da Silva, Evgeniy Svirin, Emma Sprooten, Tatyana Strekalova, ADHD Working Group of the Psychiatric Genomics Consortium, 23andMe Research team, Alejandro Arias-Vasquez, Edmund J. S. Sonuga-Barke, Philip Asherson, Claiton Henrique Dotto Bau, Jan K. Buitelaar, Bru Cormand, Stephen V. Faraone, Jan Haavik, Stefan E. Johansson, Jonna Kuntsi, Henrik Larsson, Klaus-Peter Lesch, Andreas Reif, Luis Augusto Rohde, Miquel Casas, Anders D. Børglum, Barbara Franke, Josep Antoni Ramos-Quiroga, María Soler Artigas, Marta Ribasés

**Affiliations:** 1 Psychiatric Genetics Unit, Group of Psychiatry, Mental Health, and Addiction, Vall d’Hebron Research Institute (VHIR), Universitat Autònoma de Barcelona, Barcelona, Catalonia Spain; 2 Department of Psychiatry, Hospital Universitari Vall d’Hebron, Barcelona, Catalonia Spain; 3 Department of Biomedicine (Human Genetics), and Centre for Integrative Sequencing, iSEQ, Aarhus University, Aarhus, Denmark; 4 The Lundbeck Foundation Initiative for Integrative Psychiatric Research, iPSYCH, Aarhus, Denmark; 5 Center for Genomics and Personalized Medicine, Aarhus, Denmark; 6 Biomedical Network Research Centre on Mental Health (CIBERSAM), Instituto de Salud Carlos III, Madrid, Spain; 7 Department of Genetics, Microbiology, and Statistics, Faculty of Biology, Universitat de Barcelona, Barcelona, Spain; 8 Department of Biomedicine, University of Bergen, Bergen, Norway; 9 Analytic and Translational Genetics Unit, Department of Medicine, Massachusetts General Hospital, and Harvard Medical School, Boston, MA USA; 10 Stanley Center for Psychiatric Research, Broad Institute of MIT, and Harvard, Cambridge, MA USA; 11 Department of Human Genetics, Donders Institute for Brain, Cognition, and Behaviour, Radboud University Medical Center, Nijmegen, The Netherlands; 12 University Medical Center Utrecht, UMC Utrecht Brain Center, Department of Psychiatry, Utrecht, The Netherlands; 13 ADHD Outpatient Program, Adult Division, Hospital de Clínicas de Porto Alegre, Porto Alegre, Brazil; 14 Department of Psychiatry, Donders Institute for Brain, Cognition, and Behaviour, Radboud University Medical Center, Nijmegen, The Netherlands; 15 Department of Psychiatry, Psychosomatics, and Psychotherapy, University of Würzburg, Würzburg, Germany; 16 Department of Psychiatry, Psychosomatic Medicine, and Psychotherapy, University Hospital Frankfurt, Frankfurt am Main, Germany; 17 Banc de Sang i Teixits (BST), Barcelona, Spain; 18 Grup de Medicina Transfusional, Vall d’Hebron Institut de Recerca, Universitat Autònoma de Barcelona (VHIR-UAB), Barcelona, Spain; 19 Department of Psychiatry and Legal Medicine, Universitat Autònoma de Barcelona, Barcelona, Catalonia Spain; 20 Group of Psychiatry, Mental Health, and Addiction, Vall d’Hebron Research Institute (VHIR), Barcelona, Catalonia Spain; 21 Division of Molecular Psychiatry, Center of Mental Health, University of Würzburg, Würzburg, Germany; 22 Center for Genomic Medicine, Massachusetts General Hospital, Boston, MA USA; 23 Department of Psychiatry, Harvard Medical School, Boston, MA USA; 24 Department of Psychiatry, Faculty of Medicine, Universidade Federal do Rio Grande do Sul, Porto Alegre, Brazil; 25 Division of Psychiatry, Haukeland University Hospital, Bergen, Norway; 26 Department of Genetics, Institute of Biosciences, Universidade Federal do Rio Grande do Sul, Porto Alegre, Brazil; 27 Center for Medical Genetics and Molecular Medicine, Haukeland University Hospital, Bergen, Norway; 28 Department of Clinical Science, University of Bergen, Bergen, Norway; 29 Department of Biological and Medical Psychology, University of Bergen, Bergen, Norway; 30 Department of Physiology and Biophysics, Institute of Biomedical Sciences, University of São Paulo, São Paulo, Brazil; 31 Laboratory of Psychiatric Neurobiology, Institute of Molecular Medicine, IM Sechenov First Moscow State Medical University, Moscow, Russia; 32 Department of Cognitive Neuroscience, Donders Institute for Brain, Cognition, and Behaviour, Radboud University Medical Centre, Nijmegen, The Netherlands; 33 Department of Neuroscience, School for Mental Health and Neuroscience (MHeNS), Maastricht University, Maastricht, The Netherlands; 34 Institute of Psychiatry, Psychology, and Neuroscience, King’s College London, London, UK; 35 Department of Child and Adolescent Psychiatry, Aarhus University, Aarhus, Denmark; 36 Social Genetic and Developmental Psychiatry, Institute of Psychiatry Psychology and Neuroscience, King’s College London, London, UK; 37 Karakter Child and Adolescent Psychiatry, Nijmegen, The Netherlands; 38 Institut de Biomedicina de la Universitat de Barcelona (IBUB), Barcelona, Catalonia Spain; 39 Centro de Investigación Biomédica en Red de Enfermedades Raras (CIBERER), Barcelona, Spain; 40 Institut de Recerca Sant Joan de Déu (IRSJD), Esplugues de Llobregat, Catalonia Spain; 41 Departments of Psychiatry, of Neuroscience, and Physiology, SUNY Upstate Medical University, Syracuse, NY USA; 42 School of medical Sciences, Örebro University, Örebro, Sweden; 43 Department of Medical Epidemiology and Biostatistics, Karolinska Institutet, Stockholm, Sweden; 44 Division of Child Psychiatry, Hospital de Clinicas de Porto Alegre, Federal University of Rio Grande do Sul, Porto Alegre, Brazil

**Keywords:** Genetic markers, ADHD

## Abstract

Attention deficit/hyperactivity disorder (ADHD) is a common neurodevelopmental disorder characterized by age-inappropriate symptoms of inattention, impulsivity, and hyperactivity that persist into adulthood in the majority of the diagnosed children. Despite several risk factors during childhood predicting the persistence of ADHD symptoms into adulthood, the genetic architecture underlying the trajectory of ADHD over time is still unclear. We set out to study the contribution of common genetic variants to the risk for ADHD across the lifespan by conducting meta-analyses of genome-wide association studies on persistent ADHD in adults and ADHD in childhood separately and jointly, and by comparing the genetic background between them in a total sample of 17,149 cases and 32,411 controls. Our results show nine new independent loci and support a shared contribution of common genetic variants to ADHD in children and adults. No subgroup heterogeneity was observed among children, while this group consists of future remitting and persistent individuals. We report similar patterns of genetic correlation of ADHD with other ADHD-related datasets and different traits and disorders among adults, children, and when combining both groups. These findings confirm that persistent ADHD in adults is a neurodevelopmental disorder and extend the existing hypothesis of a shared genetic architecture underlying ADHD and different traits to a lifespan perspective.

## Introduction

Attention deficit/hyperactivity disorder (ADHD) is a common neurodevelopmental disorder that severely impairs the daily functioning of patients due to age-inappropriate levels of impulsivity and hyperactivity, and/or difficulties in focusing attention [1]. ADHD has a prevalence of 5–6% in childhood, and impairing symptoms persist into adulthood in around two-thirds of children with ADHD diagnosis, with an estimated adult prevalence around 3.4% [1, 2].

ADHD is a multifactorial disorder with heritability averaging 76% throughout the lifespan [3–5]. There is consistent evidence that both common and rare variants make an important contribution to the risk for the disorder [6–11]. Several genome-wide association studies (GWAS) and meta-analyses across those have been conducted [7], but only the largest GWAS meta-analysis (GWAS-MA) performed to date reported genome-wide significant loci [6]. This study concluded that common genetic variants (minor allele frequency, MAF > 0.01) account for 22% of the heritability of the disorder [6] and supported substantial genetic overlap between ADHD and other brain disorders and behavioral/cognitive traits [6, 12].

The presentation of ADHD symptoms changes from childhood to adulthood, with lower levels of hyperactivity in adulthood but a high risk for ongoing attention problems, disorganization, and emotional dysregulation [13, 14]. As in the general population, the pattern of psychiatric and somatic comorbid conditions in ADHD also changes substantially over time, with learning disabilities, oppositional defiant disorder, and conduct disorder being more prevalent in children, and substance use disorders, social phobia, insomnia, obesity, and mood disorders becoming more pronounced in adulthood [1, 15–18]. In addition, persistent ADHD in adults is, compared with the general population (and to cases with remitting ADHD), associated with higher risk for a wide range of functional and social impairments, including unemployment, accidents, and criminal behavior [7, 19–23].

Several risk factors measured in childhood predict the persistence of ADHD symptoms into adulthood, such as the presence of comorbid disorders, the severity of ADHD symptoms, being exposed to psychosocial adversity, as well as having a high polygenic risk score (PRS) for childhood ADHD [24–28]. Twin studies suggest that both stable and dynamic genetic influences affect the persistence of ADHD symptoms [4, 5, 29, 30]. However, specific genetic factors differentiating childhood and persistent ADHD into adulthood are not well understood due to the lack of longitudinal studies. Molecular studies, including the most recent GWAS-MA of ADHD [6], have been performed in children and adults either separately or jointly [6, 31–40], but large-scale analyses comparing their genetic basis are yet to be conducted.

Given this background, we set out to study the contribution of common genetic variants to the risk for ADHD from a lifespan perspective by conducting the largest GWAS-MAs performed so far on persistent ADHD in adults (diagnosed according to DSM-IV/ICD-10 criteria) and on ADHD in childhood (that may include remittent and persistent forms of the disorder) separately and jointly. For the first time, we estimated the genetic correlation between childhood and persistent ADHD, compared their patterns of genetic correlation with other traits and disorders, assessed the effect of childhood ADHD PRSs on persistent ADHD, and explored whether individuals in which ADHD symptoms may persist into adulthood could be distinguished already in childhood using genetic data.

## Material and methods

### Sample description

A total of 19 GWAS of ADHD comprising 49,560 individuals (17,149 cases and 32,411 controls), provided by the Psychiatric Genomics Consortium (PGC), the Lundbeck Foundation Initiative for Integrative Psychiatric Research (iPSYCH), and the International Multi-centre persistent ADHD CollaboraTion (IMpACT), were analyzed. All participants were of European ancestry, had provided informed consent, and all sites had documented permission from local ethics committees.

The meta-analysis on persistent ADHD was conducted in 22,406 individuals (6,532 ADHD adult cases and 15,874 controls) using six datasets from the IMpACT consortium, two datasets from the PGC, and the adult subset from the iPSYCH cohort included in Demontis and Walters et al. [6]. The meta-analysis on ADHD in childhood included 27,154 individuals (10,617 cases and 16,537 controls), comprising two Brazilian and Spanish cohorts, seven datasets from the PGC, and the children subset from the iPSYCH cohort included in Demontis and Walters et al. [6]. All patients met DSM-IV/ICD-10 diagnostic criteria. In total, 7,086 new samples not included in Demontis and Walters et al. [6] were considered in the present study. Detailed information on each dataset is provided in Table S1 and in Supplementary Methods.

### GWAS and meta-analyses

Genotyping platforms and quality control (QC) filters for each of the datasets are shown in Table S1. Pre-imputation QC at individual and SNP level were performed using the Rapid Imputation and COmputational PIpeLIne with the default settings (https://sites.google.com/a/broadinstitute.org/ricopili/). Non-European ancestry samples, related and duplicated individuals, and subjects with sex discrepancies were excluded. Phasing of genotype data was performed using the SHAPEIT2 algorithm, and imputation for unrelated samples and trios was performed with MaCH, IMPUTE2, or MINIMAC3 (http://genome.sph.umich.edu/wiki/Minimac3) depending on software availability at the time of imputation (Table S1) [41–43]. The European ancestry panel of the 1000 Genomes Project using genome build hg19 was considered as reference for genotype imputation (ftp://ftp.1000genomes.ebi.ac.uk/vol1/ftp/). After imputation, the association with ADHD of genotype dosages was tested using logistic regression in PLINK 1.9 [44], assuming an additive genetic model and including sex, the first ten principal components, and other relevant covariates for each case-control study (Table S1). GWAS summary statistics were filtered prior to meta-analysis, excluding variants with MAF < 0.01, and imputation quality scores (INFO) ≤ 0.8. Inverse-variance weighted fixed-effects meta-analyses were conducted using METAL [45] and results were filtered by effective sample size > 70% of the total, defined as ${\mathrm{Neff}} = \frac{2}{{\left( {\frac{1}{{{\mathrm{Nca}}}}} \right) + \left( {\frac{1}{{{\mathrm{Nco}}}}} \right)}}$ [46]. The genome-wide significance threshold was set at *P* < 5.00E−08 to correct for multiple testing. Independent loci for variants exceeding this threshold were defined based on clumping using PLINK 1.9. Variants that were ±250 kb away from the index variant (variant with smallest *P* value in the region), with *P* value < 0.001, and with an estimated linkage disequilibrium (LD) of *r*^2^ > 0.2 with the index variant were assigned to a clump (*p*_1_ = 5.00E−08, *p*_2_ = 0.001, *r*^2^ = 0.2, kb = 250). Manhattan and Forest plots were generated using the “qqman” and “forestplot” R packages (3.4.4R version), respectively. The LocusZoom software [47] was used to generate regional association plots.

Details of downstream analyses for top signals identified are provided in the online supplement and include conditional analysis, Bayesian credible set analysis, and functional characterization of the significant variants.

### SNP-based heritability (SNP-*h*^2^)

The SNP-*h*^2^ was estimated by single-trait LD score regression using summary statistics, HapMap 3 LD-scores, considering default SNP QC filters (INFO > 0.9 and MAF > 0.01) and assuming population prevalence of 3.4, 5.5, and 5% for persistent ADHD, ADHD on childhood, and ADHD across the lifespan, respectively, [48]. Data of 1,113,287, 1,072,558, and 1,092,418 SNPs from the GWAS-MA of persistent ADHD, ADHD on childhood, and ADHD across the lifespan, respectively, were considered to estimate the liability-scale SNP-*h*^2^. Partitioning and enrichment of the heritability by functional categories was analyzed using the 24 main annotations (no window around the functional categories) described by Finucane et al. [49]. Statistical significance was set using Bonferroni correction (*P* < 2.08E−03).

### Gene-based and gene-set analyses

MAGMA software was undertaken for gene-based and gene-set association testing using summary data from our GWAS-MAs [50]. Variants were mapped to a gene if they were within 20 kb upstream or downstream from the gene according to dbSNP build 135 and NCBI 37.3 gene definitions. Genes in the MHC region (hg19:chr6:25-35M) were excluded from the analyses. LD patterns were estimated using the European ancestry reference panel of the 1000 Genomes Project. Gene sets denoting canonical pathways were downloaded from MSigDB (http://www.broadinstitute.org/gsea/msigdb), which integrates Kyoto Encyclopedia of Genes and Genomes (http://www.genome.jp/kegg/), BioCarta (http://www.biocarta.com/), Reactome (https://reactome.org/), and Gene Ontology (GO) (http://www.geneontology.org/) resources. Bonferroni correction (*P* < 2.77E−06 for 18,038 genes in persistent ADHD; *P* < 2.75E−06 for 18,218 genes in childhood ADHD; *P* < 2.79E−06 for 17,948 genes in ADHD across the lifespan) and 10,000 permutations were used for multiple testing correction in the gene-based and gene-set analyses, respectively.

### BUHMBOX analysis

The Breaking Up Heterogeneous Mixture Based On cross(X)-locus correlations (BUHMBOX) analysis [51] was used to test whether the genetic correlation between persistent ADHD and ADHD in childhood was driven by subgroup heterogeneity, found when there is a subset of children enriched for persistent ADHD-associated alleles. Subgroup heterogeneity was tested in each childhood dataset considering independent SNPs (*r*^2^ = 0.1, kb = 10,000) with MAF > 0.05 from the GWAS-MA of persistent ADHD using two different *P* value thresholds of *P* < 5.00E−05 (62 SNPs) and *P* < 1.00E−03 (710 SNPs). Results were meta-analyzed using the standard weighted sum of *z*-score approach, where *z*-scores are weighted by the square root of the effective sample size. The statistical power was calculated using 1,000 simulations, considering the ADHD children meta-analysis sample size, the odds ratios and risk allele frequencies from the GWAS-MA of persistent ADHD, and assuming 65% of heterogeneity proportion (π).

### Sign test

The direction of the effect of variants associated with ADHD in childhood was tested in persistent ADHD and vice versa, using strict clumping (*r*^2^ = 0.05, kb = 500, *p*_2_ = 0.5) and different *P* value thresholds (1.00E−07, 5.00E−07, 1.00E−06, 5.00E−06, 1.00E−05, 5.00E−05, 1.00E−04, and 5.00E−04). The concordant direction of effect was evaluated using a one sample test of the proportion with Yates’ continuity correction against a null hypothesis of *P* = 0.50 with the “stats” R package.

### Polygenic risk scoring

PRSs were constructed using different *P* value thresholds (*P* < 0.001, 0.05, 0.1, 0.2, 0.3, 0.4, 0.5, and 1) to select independent variants (*p*_1_ = 1, *p*_2_ = 1, *r*^2^ = 0.1, kb = 250) from the childhood GWAS-MA of ADHD and were then tested for association with persistent ADHD in each of the nine datasets, adjusting for the covariates included in the GWAS and using PRSice-2 (https://choishingwan.github.io/PRSice/). Best guess genotypes for nonambiguous strand variants present in all the persistent ADHD studies (missing rate < = 0.02) were included (*N*_SNPs_ = 32,584 for *P* =1). Results from the nine PRS analyses at each *P* value threshold were combined using inverse-variance weighted meta-analysis.

### Genetic correlation

Cross-trait LD score regression with unconstrained intercept was used to calculate genetic correlations (rg) between pairs of traits, considering HapMap3 LD-scores, markers with INFO ≥ 0.90, and excluding the MHC region (hg19:chr6:25-35M) [48]. Other ADHD datasets [6, 52] and phenotypes from the LD-hub centralized database [53] with heritability *z*-scores (observed heritability/observed standard error) >4 and with an observed heritability > 0.1 were considered (*N* = 139 out of 689 available traits). Statistical significance was set using Bonferroni correction (*P* < 3.60E−04). Pearson’s correlation coefficient (Pearson’s *r*) was calculated between the genetic correlations of persistent ADHD with the phenotypes from the LD-hub and the genetic correlations of ADHD in childhood with the phenotypes from the LD-hub.

## Results

### GWAS-MA of persistent ADHD in adults

**Fig. 1:**
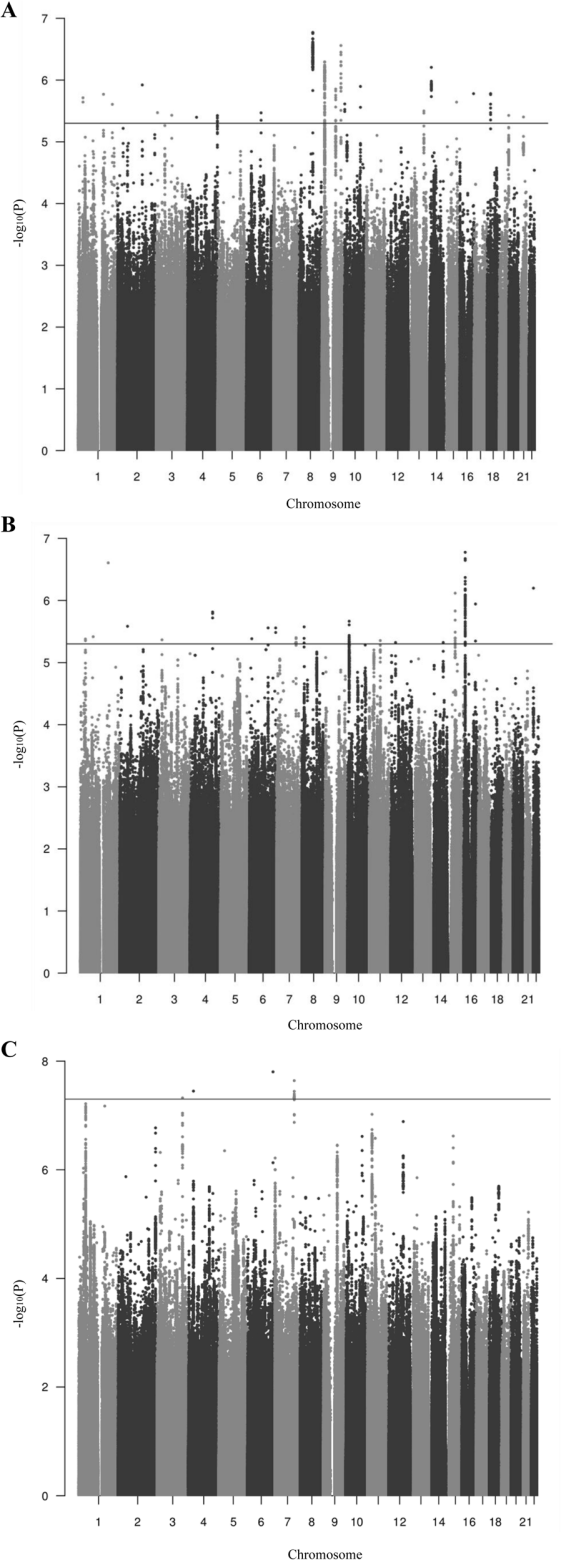
Manhattan plots of the three GWAS meta-analyses conducted. (**a**) GWAS-MA of nine cohorts of persistent ADHD in adults, (**b**) GWAS-MA of ten cohorts of ADHD in childhood, and (**c**) GWAS-MA of all datasets of ADHD across the lifespan (ADHD in childhood + persistent ADHD). Horizontal lines indicate suggestive (*P* value = 5.00E−06) and genome-wide significant (*P* = 5.00E−08) thresholds in **a**-**b**, and **c**, respectively.

The GWAS-MA of persistent ADHD in adults included 6,532 adult ADHD cases and 15,874 controls. Minimal population stratification or other systematic biases were detected (LD score regression intercept = 1.01, Fig. S1a). The proportion of heritability of persistent ADHD attributable to common single-nucleotide polymorphisms on the liability scale (SNP-*h*^2^) was 0.19 (SE = 0.024), with a nominally significant enrichment in the heritability of variants located in conserved genomic regions (*P* = 5.18E−03) and in the cell-specific histone mark H3K4me1 (*P* = 3.17E−02) (Fig. S2a). The gene-based analysis revealed six genes in four loci (*ST3GAL3, FRAT1/FRAT2, CGB1*, and *RNF225/ZNF584*) significantly associated with persistent ADHD, with *ST3GAL3* being the most significant one (*P* = 8.72E−07) (Table S2a). The single-marker analysis showed no variants exceeding genome-wide significance, with the most significant signal being rs3923931 (*P* = 1.69E−07) (Fig. 1a and Table S3a). Similarly, no significant gene sets were identified in the pathway analysis after correction for multiple comparisons (Table S4a [excel file]).

### GWAS-MA of ADHD in childhood

To compare the genetic background between persistent ADHD in adults and ADHD in childhood (that may include future remittent and persistent forms of the disorder), we conducted a GWAS-MA on children with ADHD in a total of 10,617 ADHD cases and 16,537 controls. We found no evidence of genomic inflation or population stratification (LD score regression intercept = 1.02, Fig. S1b). The liability-scale SNP-*h*^2^ for ADHD in childhood was 0.19 (SE = 0.021), with a significant enrichment in the heritability of variants located in conserved genomic regions after Bonferroni correction (*P* = 1.21E−06) (Fig. S2b). The gene-based analysis highlighted a significant association between *FEZF1* and ADHD in childhood (*P* = 5.42E−07) (Table S2b). No single genetic variant exceeded genome-wide significance, with the top signal being in rs55686778 (*P* = 1.67E−07) (Fig. 1b and Table S3b), and no significant gene sets were identified in the pathway analysis after correction for multiple comparisons (Table S4b [excel file]).

### Comparison of the genetic background of persistent ADHD in adults and ADHD in childhood

**Fig. 2:**
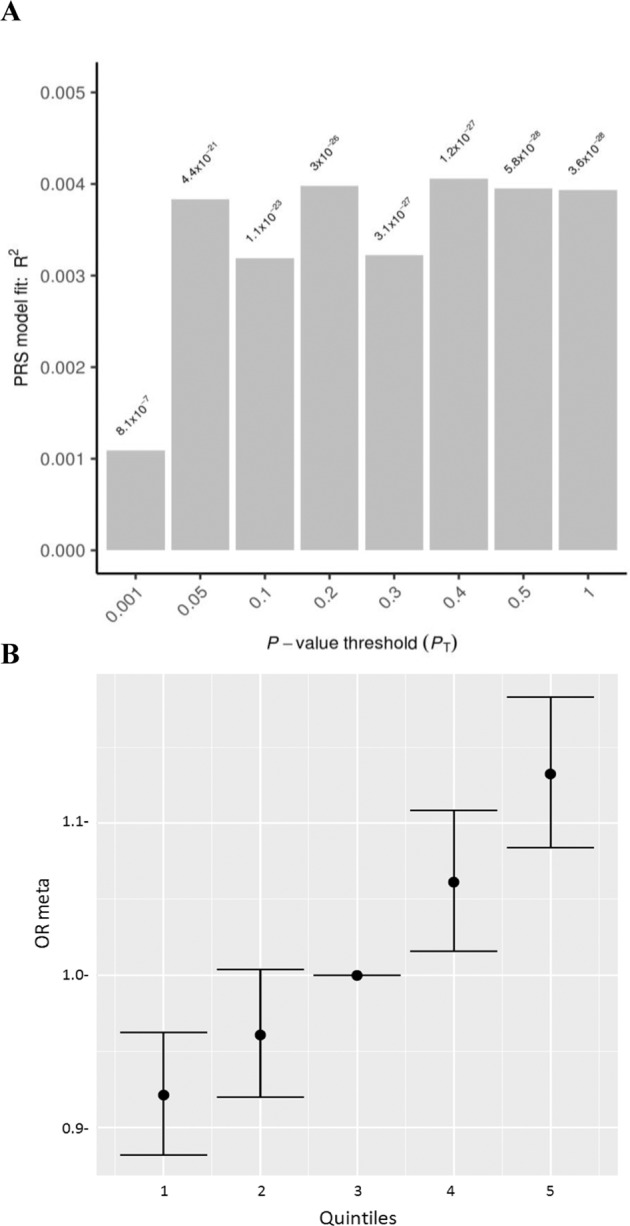
Polygenic risk scores for ADHD in childhood tested on persistent ADHD as target sample. **a** Bar plot and **b** quintile plot of meta-analysis odds ratios (OR meta) with 95% confidence intervals for *P* value threshold = 0.4 using the third quintile as baseline.

We found a strong genetic correlation between persistent ADHD in adults and ADHD in childhood (rg = 0.81, 95% CI: 0.64–0.97), significantly different from 0 (*P* = 2.13E−21) and from 1 (*P* = 0.02). Sign test results provided evidence of a consistent direction of effect of genetic variants associated with ADHD in childhood in persistent ADHD and vice versa (*P* = 6.60E−04 and *P* = 4.47E−03, respectively, for variants with *P* < 5.00E−05 in each dataset) (Table S5). In addition, PRS analyses showed that childhood ADHD PRSs were associated with persistent ADHD at different predefined *P* value thresholds, with the *P* = 0.40 threshold (*N*_SNPs_ = 20,398) explaining the most variance (*r*^2^ = 0.0041 and *P* = 1.20E−27) (Fig. 2a). The quintiles of the PRS built using this threshold showed the expected trend of higher ADHD risk for individuals in higher quintiles (Fig. 2b, Table S6).

We then tested whether the genetic correlation between persistent ADHD and ADHD in childhood was driven by a subset of children enriched for persistent ADHD-associated alleles using the Breaking Up Heterogeneous Mixture Based On Cross-locus correlations (BUHMBOX) analysis. We found no evidence of subgroup genetic heterogeneity in children, supporting that the sharing of persistent ADHD-associated alleles between children and adults was driven by the whole group of children, with a statistical power of 98.4 and 100% for thresholds of *P* < 5.00E−05 and *P* < 1.00E−03, respectively (Table S7).

### GWAS-MA of ADHD across the lifespan

**Fig. 3:**
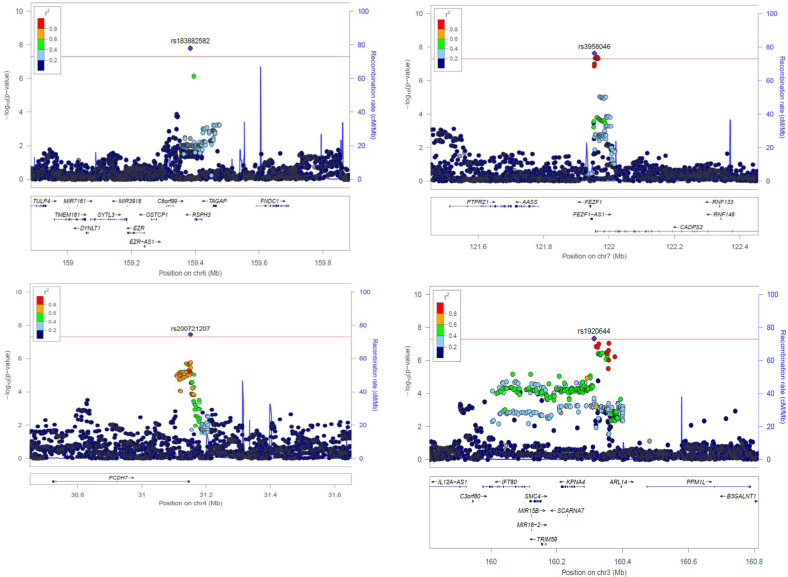
Regional association plots for genome-wide significant loci identified in the GWAS meta-analysis of ADHD across the lifespan. Each plot includes information about the locus, the location and orientation of the genes in the region, the local estimates of recombination rate (in the right corner), and the LD estimates of surrounding SNPs with the index SNP (*r*^2^ values are estimated based on 1000 Genomes European reference panel), which is indicated by color (in the upper left corner).

Given the strong genetic correlation between persistent ADHD in adults and in childhood, we performed a GWAS-MA of ADHD across the lifespan considering all datasets included in the GWAS-MAs. In total, 17,149 ADHD cases and 32,411 controls were included, and no evidence of genomic inflation or population stratification was found (LD score regression intercept = 1.03, Fig. S1c). The liability-scale SNP-*h*^2^ for ADHD across the lifespan was 0.17 (SE = 0.013), and a significant enrichment in the heritability of variants located in conserved genomic regions was observed after Bonferroni correction (*P* = 1.53E−06) (Fig. S2c). We identified four genome-wide significant variants (Figs. 1c and 3, Table 1a, and Fig. S3) and nine genes in seven loci (*FEZF1, DUSP6, ST3GAL3/KDM4A, SEMA6D, C2orf82/GIGYF2, AMN*, and *FBXL17*) significantly associated with ADHD across the lifespan (Table 1b). The most significantly associated locus was on chromosome 6 (index variant rs183882582-T, OR = 1.43 (95% CI: 1.26–1.60), *P* = 1.57E−08), followed by loci on chromosome 7 (index variant rs3958046), chromosome 4 (index variant rs200721207), and chromosome 3 (index variant rs1920644) (Table 1a, Fig. 3). The gene-set analysis showed a significant association of the “ribonucleoprotein complex” GO term with ADHD across the lifespan (*P*.adj = 0.021) (Table S4c [excel file]).

**Table 1:** Genome-wide significant loci in the GWAS meta-analysis of ADHD across the lifespan identified through (A) single-variant analysis and (B) gene-based analysis.

Chr	BP	SNP	Effect allele	Freq effect allele	OR	CI 95%	*P* value	Gene
A								
6	159384224	rs183882582	T	0.98	1.43	1.26–1.60	1.57E−08	*RSPH3* (+14 kb)
7	121955328	rs3958046	T	0.40	1.09	1.06–1.10	2.28E−08	*CADPS2* (+3.2 kb)/*FEZF1* (−13.9 kb)/*FEZF1-AS1* (+5.2 kb)
4	31151465	rs200721207	T	0.66	1.10	1.06–1.13	3.56E−08	*PCDH7* (−3.0 kb)
3	160313354	rs1920644	T	0.52	1.09	1.05–1.12	4.74E−08	*BC125159* (+27.9 kb)/*KPNA4* (−30 kb)/*ARL14* (−81.6 kb)

Gene	Chr	Start	Stop	*N* SNPs*	*N* PARAM**	Z-STAT	*P* value	
B								
*FEZF1*	7	121921373	121971173	108	18	5.6	9.57E−09	
*DUSP6*	12	89721837	89766296	103	12	5.4	3.51E−08	
*ST3GAL3*	1	44153204	44416837	521	19	5.4	3.58E−08	
*SEMA6D*	15	47456403	48086420	1565	55	5.3	7.24E−08	
*KDM4A*	1	44095797	44191189	169	13	4.9	4.34E−07	
*C2orf82*	2	233713724	233761111	138	17	4.8	7.74E−07	
*GIGYF2*	2	233542015	233745287	511	19	4.8	8.36E−07	
*AMN*	14	103368993	103417179	101	21	4.6	2.56E−06	
*FBXL17*	5	107174734	107738080	1273	35	4.6	2.59E−06	

The location (chromosome (Chr) and base position (BP)), effect allele and its frequency, odds ratio (OR) of the effect allele with 95% confidence interval (CI 95%) and association *P* values, along with genes in the locus are shown for each index variant ID (SNP). For the gene-based results, the number of single-nucleotide polymorphisms in the genes (*) and the number of relevant parameters used in the model by MAGMA software (**) are given.

One of the four loci identified in the single-variant analysis also reached genome-wide significance in the previous GWAS-MA on ADHD [6], and all of them showed consistent direction of the effect in that study (Table S8a). Significant loci reported by Demontis et al. [6] showed nominal association with ADHD across the lifespan in our study (Table S8b, c), with single variant hits showing the same direction of the effect (Table S8b).

Analyses conditioning on the index variant for the four ADHD-associated loci did not reveal new independent markers. These four significant loci were functionally characterized by obtaining Bayesian credible sets and searching for expression quantitative trait loci (eQTL) using available data in blood or brain [54, 55]. We found that credible sets for three of the four loci contained at least one eQTL within 1 Mb of the index variant. The credible set on chromosome 6 included the index variant (rs183882582) and rs12197454. This variant, in LD with the index variant (*r*^2^ = 0.56), was associated with the expression of *RSPH3* in blood and brain (*P*.adj < 1.65E−05 and *P*.adj = 2.36E−07, respectively), and with the expression of *VIL2* in blood (*P*.adj = 3.21E−03). The credible set for the second most associated locus on chromosome 7 included 24 variants. The index variant, rs3958046, and other variants in this set, were eQTLs for *CADPS2* in brain (maximum *P*.adj = 2.91E−03). The credible set for the locus on chromosome 4 contained 50 variants, most of them located in or near *PCDH7*, but no eQTLs were identified. In the credible set for the locus on chromosome 3, which included 98 variants, the index variant, rs1920644, was associated with the expression of *KPNA4*, *IFT80*, and *KRT8P12* in brain (*P*.adj = 1.16E−04, *P*.adj = 1.40E−03, and *P*.adj = 1.77E−03, respectively). Many other variants in this set were eQTLs for these genes and also for *TRIM59*, *OTOL1*, and/or *C3orf80* in brain (*P*.adj < 0.05) (Table S9 [excel file]).

In a summary-data-based Mendelian randomization (SMR) analysis, we used summary data from the GWAS-MA of ADHD across the lifespan and the eQTL data in blood and brain from Westra et al. [54] and Qi et al. [55] to identify gene expression levels associated with ADHD. We found a significant association between ADHD across the lifespan and *RMI1* expression in blood (*P*_SMR_ = 5.36E−06) (Table S10 [in excel]), finding not likely to be an artifact due to LD between eQTL and other ADHD-associated variants given that the *P*_HEIDI_ was 0.47.

### Genetic correlation with other ADHD datasets and phenotypes

We found significant genetic correlations of ADHD in children and adults from the previous GWAS-MA [6] (*N* = 53,296) and persistent ADHD (rg = 0.85, SE = 0.04, *P* = 5.49E−99), ADHD in childhood (rg = 0.99, SE = 0.03, *P* = 5.02E−273), and ADHD across the lifespan (rg = 0.98, SE = 0.01, *P* < 2.23E−308) (Table S11). When removing sample overlap (LD score genetic covariance intercept = 0.75) and considering only the subset of new samples included in our GWAS-MA on ADHD across the lifespan (*N* = 7086), a significant genetic correlation was also obtained between their sample and ours (rg = 0.91, SE = 0.35, *P* = 8.70E−03).

We also observed significant genetic correlations between childhood ADHD symptom scores from a GWAS-MA in a population of children reported by the EAGLE consortium [52] (*N* = 17,666) and persistent ADHD (rg = 0.65, SE = 0.20, *P* = 1.10E−03), ADHD in childhood (rg = 0.98, SE = 0.21, *P* = 2.76E−06), and ADHD across the lifespan (rg = 0.87, SE = 0.19, *P* = 4.80E−06). Similarly, significant genetic correlations between GWAS of self-reported ADHD status from 23andMe (*N* = 952,652) and persistent ADHD (rg = 0.75, SE = 0.05, *P* = 2.49E−45), ADHD in childhood (rg = 0.63, SE = 0.05, *P* = 1.39E−42), and ADHD across the lifespan (rg = 0.72, SE = 0.04, *P* = 4.86E−88) were observed (Table S11).

**Fig. 4:**
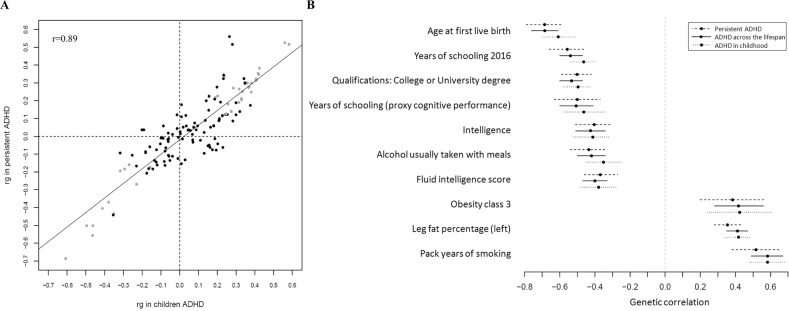
Genetic correlation of ADHD and several traits. **a** Dots represent genetic correlations (rg) for all traits considered (with *h*^2^ > 0.1 and *z*-score > 4) and those traits that met Bonferroni correction in both children and adult ADHD groups are presented in grey. *r* indicates Pearson’s correlation coefficient. **b** The ten strongest genetic correlations (with 95% confidence intervals) surpassing Bonferroni corrections in the children and persistent ADHD analysis are shown for each trait and ADHD.

We also estimated the genetic correlation of persistent ADHD in adults, ADHD in childhood, and ADHD across the lifespan with all available phenotypes in LD-hub. Results for 139 phenotypes passed the QC parameters and 41 genetic correlations were significant after Bonferroni correction in both children and adults with persistent ADHD (Table S12 [excel file]). Again, the genetic correlations with ADHD were consistent across the lifespan, with similar patterns found in adulthood and childhood (Pearson’s *r* = 0.89) (Fig. 4a, Table S12 [excel file]). The strongest genetic correlations with ADHD were found for traits related to academic performance, intelligence, and risk-taking behaviors, including smoking and early pregnancy (Fig. 4b).

## Discussion

In the current study, we set out to explore the contribution of common genetic variants to the risk of ADHD across the lifespan by conducting GWAS-MAs separately for children and adults with persistent ADHD that meet DSM-IV/ICD-10 criteria. Using the largest GWAS datasets available from the PGC, the iPSYCH, and IMpACT consortia we found evidence for a common genetic basis for ADHD in childhood and persistent ADHD in adults and identified nine new loci associated with the disorder.

We found a highly similar proportion of the heritability of ADHD explained by common variants in children and in adults (SNP-*h*^2^ = 0.19), which is consistent with the SNP-*h*^2^ estimate reported in the recent GWAS-MA on ADHD [6] (SNP-*h*^2^ = 0.22), that included children and adults, and is in line with multiple studies supporting the stability of ADHD’s heritability from childhood to adulthood [3–5]. These results together with the 0.81 genetic correlation found between children and adults with persistent ADHD reinforce the hypothesis of the neurodevelopmental nature of persistent ADHD in adults. Consistently, the sign test and the PRS analysis confirmed the extensive overlap of common genetic risk variants for ADHD in childhood and adulthood.

In the view of the fact that children with ADHD may be an admixed group of individuals whose ADHD symptoms will persist or remit in adulthood, we ran a BUHMBOX analysis to elucidate if the potential “persistent” individuals could be distinguishable already in childhood. Our data supported genetic similarities in ADHD across the lifespan with no evidence of a subset of patients enriched for persistent ADHD-associated alleles within the group of children.

Despite not having identified specific genetic contributions for ADHD in children or persistent ADHD, our results are not inconsistent with evidence suggesting changes in the genetic contribution to ADHD symptoms from childhood into adulthood, as described in previous twin studies in the general population [4, 5, 29, 30]. Our study design and the still limited statistical power of the GWAS-MAs may have facilitated the identification of the shared genetic basis rather than specific genetic factors for persistence. Also, differences between the origin of the samples (population-based versus clinical) and/or discrepancies between self- and medical reports could explain why we found no group-specific genetic variants. In addition, given that Chen et al. [56] and Biederman et al. [57] reported that persistence of ADHD into adulthood indexed stronger familial aggregation of ADHD, we cannot yet discard influences of non-additive genetic effects, or other types of genetic variation, such as rare mutations or copy number variation, playing a role in the different ADHD trajectories across the lifespan.

We also found strong and significant positive genetic correlations of ADHD ascertained in clinical populations of adults, children, or both with other ADHD-related measures from general population samples, including the largest GWAS of self-reported ADHD status from 23andMe participants (*N* = 952,652) and the GWAS-MA of childhood rating scales of ADHD symptoms in the general population [52]. In agreement with previous reports, these data suggest that a clinical diagnosis of ADHD in adults is an extreme expression of continuous heritable traits [6] and that a single question about ever having received an ADHD diagnosis, as in the 23andMe sample, may be informative for molecular genetics studies.

Similar patterns of genetic correlation of ADHD with different somatic disorders and anthropometric, cognitive, and educational traits were identified for children and adults. These findings were highly similar to those observed in the recent GWAS-MA [6] and further extend the existing hypothesis of a shared genetic architecture underlying ADHD and these traits to a lifespan perspective.

We report 13 loci in gene- and SNP-based analyses for childhood ADHD, adult ADHD, and/or ADHD across the lifespan. Four ADHD-associated loci were previously identified by Demontis et al. [6], which was expected due to the sample overlap between the two datasets. The new loci identified in the present study mainly included genes involved in brain formation and function, such as *FEZF1*, a candidate for autism spectrum disorder implicated in the formation of the diencephalon [58, 59], *RSPH3*, which participates in neuronal migration in embryonic brain [60], *CADPS2*, which has been associated with psychiatric conditions due to its role in monoamine and neurotrophin neurotransmission [61–64], *AMN*, which is involved in the uptake of vitamin B12 [65, 66], essential for brain development, neural myelination, and cognitive function [67], and *FBXL17*, which has previously been related to intelligence [68].

The main limitation of this study is the sample overlap (85.7%) between the present GWAS-MAs and the previous one by Demontis et al. [6], which highlighted loci previously associated with ADHD. Although sample overlap may have inflated the genetic correlation found between these studies, the estimate remained strong and significant when excluding nonoverlapping datasets.

In summary, the present cross-sectional analyses identify new genetic loci associated with ADHD and, more importantly, support the hypothesis that persistent ADHD in adults is a neurodevelopmental disorder that shows a high and significant genetic overlap with ADHD in children. Future longitudinal studies will be required to disentangle the role of common genetic variants on ADHD remittance and/or persistence.

## Funding and disclosure

VR has served on the speakers for Eli Lilly, Rubio, and Shire in the last 5 years. She has received travel awards from Eli Lilly and Co. and Shire for participating in psychiatric meetings. The ADHD Program has received unrestricted educational and research support from Eli Lilly and Co., Janssen-Cilag, Shire, Rovi, Psious, and Laboratorios Rubió in the past 2 years. MC received travel awards for taking part in psychiatric meetings from Shire. CF received travel awards for taking part in psychiatric meetings from Shire and Lundbeck. GEM received travel awards for taking part in psychiatric meetings from Shire. EJSS-B received speaker fees, consultancy, research funding, and conference support from Shire Pharma. Consultancy from Neurotech Solutions, Copenhagen University and Berhanderling, Skolerne & KU Leuven. Book royalties from OUP and Jessica Kingsley. Financial support—Arhus University and Ghent University for visiting Professorship. Editor-in-Chief JCPP—supported by a buy-out of time to University of Southampton and personal Honorarium. King’s College London received payments for work conducted by PA: consultancy for Shire, Eli Lilly, Novartis, and Lundbeck; educational and/or research awards from Shire, Eli Lilly, Novartis, Vifor Pharma, GW Pharma, and QbTech; speaker at events sponsored by Shire, Eli Lilly, Janssen-Cilag, and Novartis. JKB has been in the past 3 years a consultant to/member of advisory board of and/or speaker for Shire, Roche, Medice, and Servier. He is not an employee of any of these companies, and not a stock shareholder of any of these companies. He has no other financial or material support, including expert testimony, patents, royalties. In the past year, SVF received income, potential income, travel expenses continuing education support and/or research support from Tris, Otsuka, Arbor, Ironshore, Shire, Akili Interactive Labs, Enzymotec, Sunovion, Supernus, and Genomind. With his institution, he has US patent US20130217707 A1 for the use of sodium–hydrogen exchange inhibitors in the treatment of ADHD. He also receives royalties from books published by Guilford Press: Straight Talk about Your Child’s Mental Health, Oxford University Press: Schizophrenia: The Facts and Elsevier: ADHD: Non-Pharmacologic Interventions. He is principal investigator of www.adhdinadults.com. JK has given talks at educational events sponsored by Medice; all funds are received by King’s College London and used for studies of ADHD. HL has served as a speaker for Evolan Pharma and Shire and has received research grants from Shire; all outside the submitted work. K-PL served as a speaker for Eli Lilly and received research support from Medice, and travel support from Shire, all outside the submitted work. LAR reported receiving honoraria, serving on the speakers’ bureau/advisory board, and/or acting as a consultant for Eli Lilly, Janssen-Cilag, Novartis, and Shire in the last 3 years; receiving authorship royalties from Oxford Press and ArtMed; and receiving travel awards from Shire for his participation in the 2015 WFADHD meetings and from Novartis to take part of the 2016 AACAP meeting. The ADHD and juvenile bipolar disorder outpatient programs chaired by him received unrestricted educational and research support from the following pharmaceutical companies in the last 3 years: Janssen-Cilag, Novartis, and Shire. MC has received travel grants and research support from Eli Lilly and Co., Janssen-Cilag, Shire, and Lundbeck and served as consultant for Eli Lilly and Co., Janssen-Cilag, Shire, and Lundbeck. BF has received educational speaking fees from Medice and Shire. JAR-Q was on the speakers’ bureau and/or acted as consultant for Eli Lilly, Janssen-Cilag, Novartis, Shire, Lundbeck, Almirall, Braingaze, Sincrolab, Medice, and Rubió in the last 5 years. He also received travel awards (air tickets + hotel) for taking part in psychiatric meetings from Janssen-Cilag, Rubió, Shire, Medice, and Eli- Lilly. The Department of Psychiatry chaired by him received unrestricted educational and research support from the following companies in the last 5 years: Eli Lilly, Lundbeck, Janssen- Cilag, Actelion, Shire, Ferrer, Oryzon, Roche, Psious, and Rubió. The other authors have nothing to disclose. All members of the 23andMe Research Team are current or former employees of 23andMe, Inc. and hold stock or stock options in 23andMe. Authors of the ADHD Working group of the PGC that participated in this study: Catharina A. Hartman, Ziarih Hawi, Jennifer Crosbie, Sandra Loo, Josephine Elia, Russell Schachar, Christie Burton, Ted Reichborn-Kjennerud, Aribert Rothenberger, Søren Dalsgaard, Irwin Waldman, Mark Bellgrove, Hakon Hakonarson, Johannes Hebebrand, Anke Hinney, and Robert Oades have nothing to disclose. Joseph Biederman 2019–2020: he received research support from Genentech, Headspace Inc., Lundbeck AS, Neurocentria Inc, Pfizer Pharmaceuticals, Roche TCRC Inc., Shire Pharmaceuticals Inc., Sunovion Pharmaceuticals Inc., and Tris. He was a consultant for Akili, Avekshan, Jazz Pharma, and Shire/Takeda. Through MGH CTNI, he participated in a scientific advisory board for Supernus. Dr Biederman’s program has received departmental royalties from a copyrighted rating scale used for ADHD diagnoses, paid by Bracket Global, Ingenix, Prophase, Shire, Sunovion, and Theravance; these royalties were paid to the Department of Psychiatry at MGH. Tobias Banaschewski served in an advisory or consultancy role for Lundbeck, Medice, Neurim Pharmaceuticals, Oberberg GmbH, Shire, and Infectopharm. He received conference support or speaker’s fee by Lilly, Medice, and Shire. He received royalties from Hogrefe, Kohlhammer, CIP Medien, and Oxford University Press. James McGough is an expert testimony for Eli Lilly; DSMB for Sunovion. Benjamin Neale is a member of the scientific advisory board at Deep Genomics and consultant for Camp4 Therapeutics, Takeda Pharmaceutical, and Biogen. Ole Andreas Andreassen received speaker’s honorarium from Lundbeck, and is a consultant for HealthLytix.

The research leading to these results has received funding from the European Community’s Seventh Framework Programme (FP7/2007–2013) under grant agreement n° 602805 (Aggressotype) as well as from the European Union H2020 Programme (H2020/2014–2020) under grant agreements n° 643051 (MiND), n° 667302 (CoCA), n° 728018 (Eat2beNICE), and n° 278948 (TACTICS). The work was also supported by the ECNP Network “ADHD across the Lifespan” (https://www.ecnp.eu/research-innovation/ECNP-networks/List-ECNP-Networks/ADHD.aspx). Over the course of this investigation, PR is a recipient of a pre-doctoral fellowship from the Agència de Gestió d’Ajuts Universitaris i de Recerca (AGAUR), Generalitat de Catalunya, Spain (2016FI_B 00899). The iPSYCH project is funded by the Lundbeck Foundation (grant nos. R102-A9118 and R155-2014-1724) and the universities, and university hospitals of Aarhus and Copenhagen. ADB and NRM’s work is also supported by the EU’s Horizon 2020 programme (grant no. 667302, CoCA). Data handling and analysis was supported by NIMH (1U01MH109514-01 to Michael O’Donovan and ADB). CSM is a recipient of a Sara Borrell contract from the Instituto de Salud Carlos III, Ministerio de Economía, Industria y Competitividad, Spain (CD15/00199). K-PL and his team are supported by the Deutsche Forschungsgemeinschaft (DFG: CRU 125, CRC TRR 58 A1/A5, no. 44541416), ERA-Net NEURON/RESPOND, no. 01EW1602B, ERA-Net NEURON/DECODE, no. 01EW1902, and 5-100 Russian Academic Excellence Project. JH thanks Stiftelsen K.G. Jebsen, University of Bergen, the Western Norwegian Health Authorities (Helse Vest). HL thanks the Swedish research council. BC received financial support from the Spanish “Ministerio de Economía y Competitividad” (SAF2015-68341-R) and AGAUR (2017SGR738). MSA is a recipient of a contract from the Biomedical Network Research Center on Mental Health (CIBERSAM), Madrid, Spain. MR is a recipient of a Miguel de Servet contract from the Instituto de Salud Carlos III, Spain (CP09/00119 and CPII15/00023). The research leading to these results has received funding from the Instituto de Salud Carlos III (PI16/01505, PI17/00289, PI18/01788, PI19/00721 and P19/01224), and cofinanced by the European Regional Development Fund (ERDF), from the Pla estratègic de recerca i innovació en salut (PERIS); Generalitat de Catalunya (METAL-Cat; SLT006/17/287), from “la Marató de TV3” (092330/31) and from the Agència de Gestió d’Ajuts Universitaris i de Recerca-AGAUR, Generalitat de Catalunya (2017SGR1461). ES’s work is supported by a personal Hypatia grant from the Radboud University Medical Center. MH received a Veni grant from of the Netherlands Organization for Scientific Research (NWO, grant number 91619115). The NeuroIMAGE study was supported by NIH Grant R01MH62873 (to SVF), NWO Large Investment Grant 1750102007010 (to JKB), ZonMW grant 60-60600-97-193, NWO grants 056-13-015 and 433-09-242, and matching grants from Radboud University Nijmegen Medical Center, University Medical Center Groningen and Accare, and Vrije Universiteit Amsterdam. Organization for Scientific Research (NWO; grant 016-130-669). BF and MK’s work is supported by the Dutch National Science Agenda (NWA) for the NeurolabNL project (grant 400-17-602). This paper represents independent research part funded by the National Institute for Health Research (NIHR) Biomedical Research Centre at South London and Maudsley NHS Foundation Trust and King’s College London. BF received additional funding from a personal Vici grant of the Dutch. The authors declare no competing interests

## Supplementary information

Supplementary material

Supplementary tables
